# Environmental impact of the explosion of the Nord Stream pipelines

**DOI:** 10.1038/s41598-023-47290-7

**Published:** 2023-11-14

**Authors:** Hans Sanderson, Michał Czub, Jaromir Jakacki, Sven Koschinski, Jakob Tougaard, Signe Sveegaard, Torsten Frey, Patrik Fauser, Jacek Bełdowski, Aaron J. Beck, Anna Przyborska, Adam Olejnik, Bogdan Szturomski, Radoslaw Kicinski

**Affiliations:** 1 Department of Environmental Science, Aarhus University, 399 Frederiksborgvej, 4000 Roskilde, Denmark; 2 Department of Hydrobiology, Faculty of Biology, Institute of Functional Biology and Ecology, University of Warsaw, Warsaw, Poland; 3 Institute of Oceanology, Polish Academy of Sciences, Sopot, Poland; 4 Meereszoologie, Kühlandweg 12, 24326 Nehmten, Germany; 5 Department of Ecoscience, Aarhus University, Aarhus, Denmark; 6 GEOMAR Helmholtz Centre for Ocean Research Kiel, Kiel, Germany; 7 Faculty of Mechanical and Electrical Engineering, Polish Naval Academy, Gdynia, Poland

**Keywords:** Environmental sciences, Natural hazards, Ocean sciences

## Abstract

Armed conflicts have, in addition to severe impacts on human lives and infrastructure, also impacts on the environment, which needs to be assessed and documented. On September the 26th 2022, unknown perpetrators deliberately ruptured the two gas pipelines Nord Stream 1 and 2 with four coordinated explosions near a major chemical munition dump site near the Danish island of Bornholm in the Baltic Sea. While the massive release of natural gas into atmosphere raised serious concerns concerning the contribution to climate change—this paper assesses the overlooked direct impact of the explosions on the marine ecosystem. Seals and porpoises within a radius of four km would be at high risk of being killed by the shockwave, while temporary impact on hearing would be expected up to 50 km away. As the Baltic Proper population of harbour porpoises (*Phocoena phocoena*) is critically endangered, the loss or serious injury of even a single individual is considered a significant impact on the population. The rupture moreover resulted in the resuspension of 250000 metric tons of heavily contaminated sediment from deep-sea sedimentary basin for over a week, resulting in unacceptable toxicological risks towards fish and other biota in 11 km^3^ water in the area for more than a month.

## Introduction

On September the 26th 2022 unknown perpetrators deliberately ruptured the two gas pipelines Nord Stream 1 and Nord Stream 2 with four coordinated explosions in the Danish and Swedish exclusive economic zones (EEZ) near the Danish Island of Bornholm in the Baltic Sea. It is estimated that more than 115,000 tons natural gas (CH_4_) were released over the course of six days and contributed greenhouse gas emissions comparable to approximately 15 million tons of CO_2_—or one third of the Danish total CO_2_ annual emissions) thus contributing to global warming^1^. Other, local environmental impacts have not yet been assessed.

Aerial photos illustrate the major environmental disturbance, as the largest area of turbulence visible at the sea surface had a diameter of 1 km, according to the Danish Defense surveillance results. The explosions took place near a known Second World War-era chemical weapons dumpsite, where 11.000 tons of chemical warfare agents (CWA) were sea-dumped in 1947, causing environmental concerns in regard to CWA release^2,3^.

Three species of marine mammals are found near the explosion sites: Grey seal (*Halichoerus grypus*), harbour seal (*Phoca vitulina vitulina*) and harbour porpoise (*Phocoena phocoena*). Of these species the harbour porpoise is considered the most vulnerable in relation to explosions^4^. Furthermore, its population in the Baltic Proper (of the Baltic Sea) is very small and critically endangered^5^. Damages from explosions generally seen in harbour porpoises include tissue damage in middle ear cavities, fracture of ossicles and bleeding in the inner ear and acoustic fats of the melon and lower jaw^6^, all structures with critical function in echolocation. Sub-lethal effects also include permanent or temporary threshold shift in their hearing^4^.

This paper explores the environmental impacts of the pipeline explosions and release of gas by modelling potential impacts on marine ecosystem of the explosions themselves as well as toxic risk from resuspended contaminated seabed sediments in the water column towards biota.

## Materials and methods

### Explosion

Seismometer data suggest charge sizes with a relative effectiveness of 500 kg TNT-equivalent each were used at the explosion sites near both twin-pipelines. The north-eastern explosions near Nord-Stream 1 (NS1) took place within the deepest part of the Bornholm Basin, known as the Bornholm Deep. The south-eastern explosions near Nord-Stream 2 (NS2) took place just south of the munitions dump site. The Bornholm CWA dumpsite is located approximately 22 km north of explosion 1 and 21 km south of the explosions. The escaping gas from the exploded pipelines resulted in a pressure drop from 115 to 7 bars in the pipes indicating a significant jet of gas for six days before the pipes were emptied^1^. This in combination with the explosions were a major disturbance for local hydrodynamic generating currents that exceeded the resuspension thresholds for the seabed sediments.

We found no evidence that the natural gas release alone would cause any lasting significant marine impacts. We also assessed the relevance of explosive residues from the TNT, but assuming complete detonation, explosive chemical residues would be minimal and represent negligible toxic risk. Therefore, these two impacts are only covered in Supporting Information (SI 1).

### Description of site

The Bornholm Deep is the deepest part of the sedimentary Bornholm Basin, characterized by strong stratification in the water column with near-bottom currents rarely exceeding sediment resuspension thresholds^7^. Depths up to 100 m fall below the halocline/pycnocline, and surface sediments are dominated by muds and muddy sands affected by almost permanent hypoxia or anoxia^8^. Vast areas of the Bornholm Basin are covered with a “fluffy layer” from a constant accumulation of settling organic flocs formed in the water column above the pycnocline. These muds (the fine fraction of sediments < 2 µm) act as a major sink of pollutants due to their large surface area, abundance of clay minerals and associated coatings of organic matter and iron/manganese oxides and oxyhydroxides^9^. Therefore, a major part of heavy metals and organic contaminants contained within the bottom sediments will be associated with this fraction in the upper 25 cm of the sediment in the parts of the Baltic Sea where the accumulation happens, e.g. in the Bornholm Deep, which at a local sedimentation rate of 0.52 ± 0.02 up to 0.82 ± 0.10 mm yr^−1^ were building up throughout the whole Anthropocene^10,11^.

The detonations took place approximately 20 km away from the designated CWA dumpsite. Within a 20 kms radius zone of the explosions of the Nord Stream 1 pipeline, 39 samples containing measurable CWA levels have been recorded^12^. No samples were collected closer than 20 kms to the explosion of Nord Stream 2 pipeline. As with other pollutants, the highest CWA concentration was found in the upper top 5 cm of the collected sediments^13^. There are several different CWA residue compounds originating from either mustard gas or arsenic-based CWAs. In this assessment we combine the compounds into two classes with two mean values used in the assessment (Table 1). We assume conservatively that the sediment near the explosion sites contained the mean CWA residue concentration of these samples, despite they are not recent.

**Table 1:** Sediment concentration of CWA near the Explosions in the Bornholm Deep.

Compound	CWA concentration (µg/kg)			
	*n*	Mean	Maximum	Std. Dev
Mustard related CWAs				
1,4-Dithiane	11.0	3.1	32.0	9.6
1-oxa-4,5-dithiepane	5.0	5.4	24.0	10.5
1,2,5-Trithiepane	19.0	0.7	2.8	0.6
Sum mustard related	39.0	**9.2**	56.0	13.8
As based CWAs				
Adamsite	1.0	27.0	27.0	0
5,10-Dihydrophenoarsazin-10-ol 10-oxide	6.0	1.0	6.1	2.5
Clark I	2.0	1641.3	3262.8	2293.2
Diphenylarsenic acid	6.0	12.3	68.0	27.4
Diphenylpropylthioarsine	6.0	8.0	27.0	11.9
Triphenyl arsine	8.0	44.0	172.3	76.6
Triphenyl arsine oxide	8.0	6.9	43.6	15.1
Phenylarsonic acid	7.0	1.7	6.0	2.9
Dipropyl phenylarsonodithioite	14.0	5.2	41.0	10.7
Sum arsenic-based	39.0	**129.2**	3461.6	550.4

Significant values are in bold.

Pollutants in Baltic Sea sediments have been monitored for several decades by HELCOM Contracting Parties^14^. The following contaminants have been prioritized for the Bornholm Basin: Mercury (Hg), polybrominated diphenyl ethers (PBDEs), hexabromocyclododecanes (HBCDs), Caesium-137, anthracene, cadmium (Cd), lead (Pb), and tributyltin (TBT). Table 2 contains the mean sediment concentration of the prioritized problematic contaminants in the Bornholm Deep reported by HELCOM and European Environmental Quality Standards (EQS) for “prioritized substances and certain other pollutants” in “other surface waters” are used^15^ (Table 2). In the absence of European EQS values, national Danish EQS values are applied^16^. The toxicity of arsenic-based CWAs is evaluated according to inorganic or organic As toxicity. For mustard gas and its degradation products the lowest measured toxicity threshold value is used^17^.

**Table 2:** Mean sediment concentrations of pollutants in the Bornholm Deep^14^ with estimations of their resuspended tonnage, EQS and relative mixture toxicity contributions.

Compound	Mean concentration in Bornholm deep sediments [µg/kg]	Resuspended pollutant mass [Kg]	EQS [µg/L]	Toxic contributions [%]
Organics				
PBDE	0.31	< 0.1	0.0049	0.04
HBCD	0.92	0.2	0.0008	0.67
Anthracene	6.3	1.6	0.1	0.04
Metals				
Hg	60	15	0.05	0.7
MeHg	0.375	< 0.1	0.0065	0.03
Cd	1140	285	0.2	3.3
Pb	57,700	14,425	1.3	25.9
Cs-137	77 Bq/kg	NA	0.015 Bq/L	3.0
Cr	14,700	3675	3.4	2.5
Cu	15,500	3875	1	9.0
Zn	14,600	3650	7.8	1.1
Ni	4000	1000	8.6	0.3
As	1800	450	0.6	1.8
TBT	17.7	4.4	0.0002	51.6
Chemical warfare agents				
Sum As based	129.2	32.3	0.6	0.1
Sum mustard gas related	9.2	2.3	830	0.00001

### Marine mammals impact assessment

The range at which there was a risk of blast injury was estimated by equations provided by Yelverton et al.^18^ in the SI Fig. 1. Yelverton (1972) is of course not a recent study but it is nonetheless still relevant and valid in this screening. These equations provide estimates of the acoustic energy (acoustic impulse) as function of the size of the charge and the distance from the explosion. Impact ranges were found when the distance at which the acoustic impulse dropped to 30 µPa·s. This threshold was provided by Lance et al.^19^ and corresponds to less than 10% probability of injury to lungs or intestines in human divers. The range within which there was a risk of damage to the inner ear (acoustic trauma) was estimated based on measurements of the sound exposure level from explosions of different charge sizes up to 295 kg at different ranges for detonations of unexploded ordnance (UXO) in the North Sea^20^ and extrapolated these out to distances where the levels exceeded the thresholds for onset of noise induced permanent threshold shift (PTS)^4^. The maximum range at which harbour porpoises were susceptible to PTS was extrapolated by linear regression from the abovementioned results in the range 1–200 kg. Seals, however, are considered less susceptible to PTS at low frequencies than baleen whales^4^. Maximum impact ranges for seals were therefore estimated by correcting the results for baleen whales for both the lower frequency weighting levels and lower sensitivity of seals to PTS compared to baleen whales^20^. The ranges within which temporary threshold shift (TTS) could have occurred was estimated from the propagation loss curves^21^, by identifying the distance from the explosion where the received sound exposure level was 15 dB lower than the level at the maximum range for PTS^4^. For further information see the SI section 3.

### Sediment resuspension

During an underwater explosion sediment containing residues pollutants is resuspended into the water column dependent upon the location of the charge (explosion on the surface, below or above the ground), as well as the density and type of seabed. The volume of ejected sediment from a 500 kg load of TNT, assumed to have been used here, placed on the surface of the bottom sediment is approximately 13 m^3^. In addition, the created gas bubbles, moving upwards, lifts sediment into the water column at volume equal to about half of the volume of the bubble, which in the case of detonation of 500 kg of TNT is in the order of 1000 m^3^^21,22^.

The third factor that influences the resuspension of sediments is the gas leak from the damaged pipelines. Due to its buoyancy, the gas will be transported rapidly to the sea surface. The pressure in the pipeline before explosion was about 115 bar^1^. The rapid pressure drops in the pipeline due to the explosion generated a jet propagating towards the axis of the gas line.

To assess the of development of the resuspended sediment a 3D hydrodynamic model, coupled to a sediment transport model was implemented, based on the MIKE powered by DHI tools (https://www.mikepoweredbydhi.com)^23,24^. The model is based on a flexible, triangular mesh model, enabling high spatial resolution in the area of the gas pipeline explosion. The model includes various dynamically coupled modules^25^, including the mud transport MIKE 3 MT^26^ for simulation of the transport, sinking and deposition of sediment in the marine environment. The hydrodynamic part of the model has been obtained with the MIKE 3 FM HD module^27^. The influence of wind waves was also considered by using the Spectral Wave MIKE 21 FM SW module^28^. The modules were applied using operational meteorological data that were delivered to the system using an external Weather Research and Forecasting Model covering the entire Baltic Sea area^29^. For further information see the SI4 section.

### Marine toxic risk

To assess if the marine environment is at risk as a consequence of the release of pollutants associated with suspended sediment particles outlined above, the total toxic mixture risk characterization ratio (RCR) based on the data from Table 2 is calculated as:

1 $$\sum RCR = \mathop \sum \limits_{n} \frac{{Predicted\; Environmental \;Concentration \left( {PEC} \right)}}{{Predicted\; No\; Effect\; Concentration \left( {PNEC} \right) \;or \;Environmental \;Quality \;Standard \left( {EQS} \right)}}$$

Concentration additivity of the individual pollutants in Table 2 is assumed. As a conservative approach it is moreover assumed that the sediment particles taken up by biota release the entire amount of associated pollutants, which are subsequently taken up by the organism. We conservatively, furthermore disregards sorption and de-sorption of pollutants during transport in the water. Consequently, resuspended sediment particles have concentrations of pollutants corresponding to the mean values in Tables 1 and 2, at any given time and place. The predicted environmental concentration (PEC) for a pollutant is thus:

2 $$\begin{aligned} & PEC = Concentration \;in\; sediment \left( {\frac{\mu g}{{kg}}} \right) \cdot \\ & concentration \;of\; resuspended\; sediment \left( \frac{kg}{L} \right) \\ \end{aligned}$$

The resulting critical sediment concentration of resuspended sediment particles in the water is thus obtained when Eq. (1) equals 1. Inserting the values from Tables 1 and 2 in Eqs. (1) and (2), gives:

3 $$\begin{aligned} & \sum RCR = 1.0 = C_{sediment} \left( {critical} \right)\left( \frac{kg}{L} \right) \cdot \sum \left( {\frac{{Concentration \;in \;sediment \; \left( {\frac{\mu g}{{kg}}} \right)}}{{PNEC \; or \; EQS \; \left( {\frac{\mu g}{L}} \right)}}} \right) \mathop \to \limits^{{yields}} \\ & C_{sediment} \left( {critical} \right) = 5.8 \frac{mg}{L} \\ \end{aligned}$$

Hence, the toxic threshold of suspended sediments concentration (SSC) in the water column is 5.8 mg/L, above which there is a predicted marine environmental risk.

## Results

### Impacts on marine mammals

Ranges at which blast injury would have been likely in seals and harbour porpoises were estimated to be 4 km from the explosion at the sea surface and 20 km at the seabed (at 70 m depth). Damage to the hearing of seals and porpoises, in the form of permanent threshold shifts (PTS) could occur at distances up to 12 km for porpoises and about 1 km for seals. As a precautionary criterion for impact, the onset of temporary threshold shift (TTS) is sometimes used. Onset of TTS is predicted to occur 15 dB below the onset of PTS^4^. This level was predicted to occur at a range of 50 km for porpoises and 6 km for seals. Fishes in the vicinity of the blasts will of course also be directly impacted.

### Marine risks

**Figure 1:**
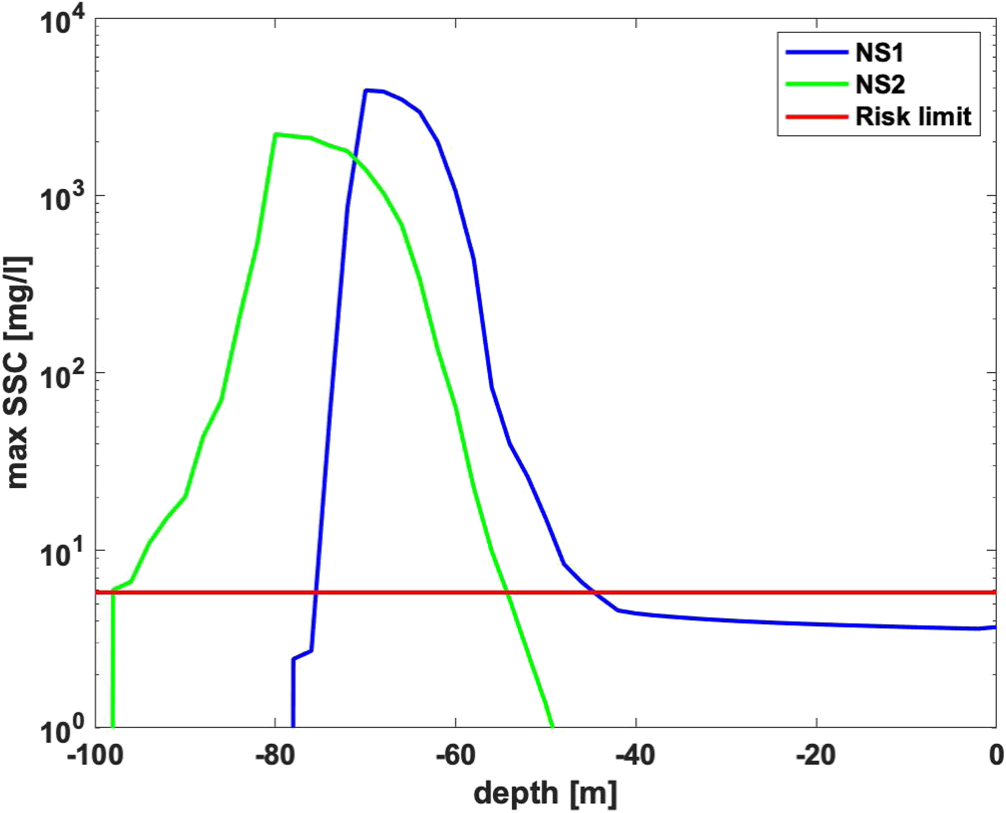
NS 1&2 maximum suspended sediments concentrations relative to depth and risk limit.

We have calculated the total amount of suspended sediments ejected into water column to be 2.5 × 10^5^ metric tons due to the explosions and the gas jet for each of the impacted areas. We found that the sediments did not reach a water depth shallower than 30 m for either of the two cases. The greatest extent from the explosion was assessed to be 26 km at a water depth of approximately 67 m from the explosion site. After approximately 35 days 50% of total suspended sediments remained in the mixed water layer. The critical threshold of suspended sediment concentration (SSC) is 5.8 mg/L at which concentration the combined mixture of contaminants represent an environmental risk was present at depths of approximately 95 m and 78 m to approximately 53 to 42 m depth, for Nord-Stream 1 & 2 (NS 1&2) respectively (Fig. 1).

**Figure 2:**
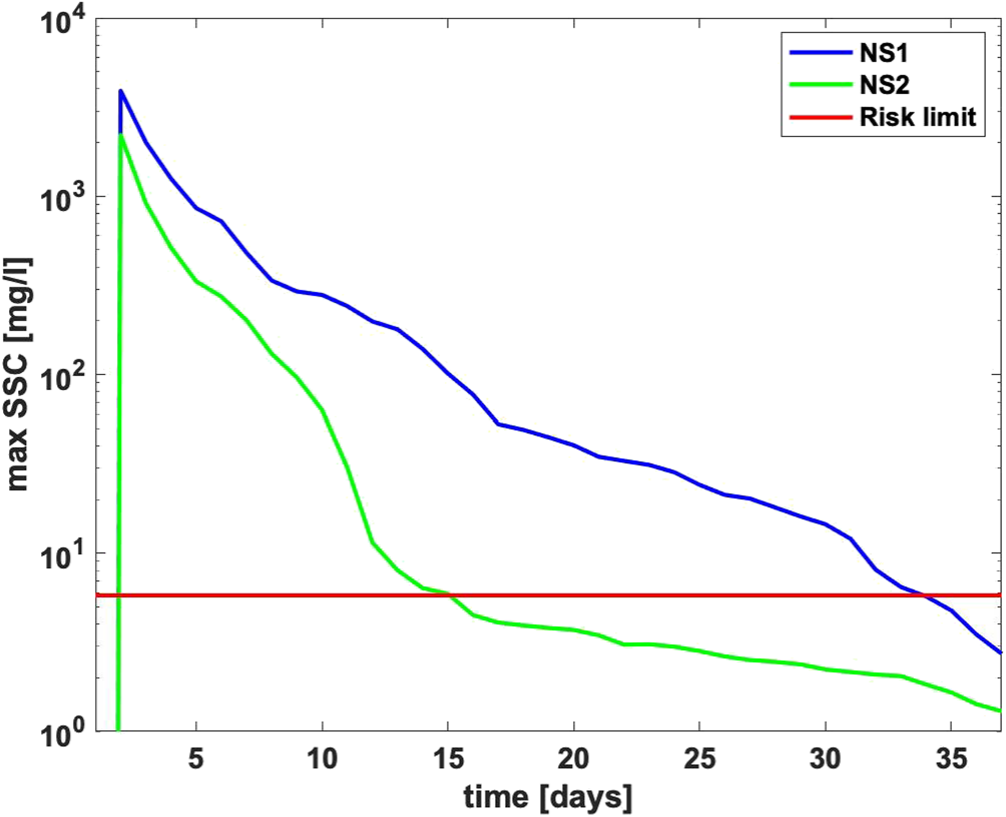
NS 1&2 duration of environmental risk.

From Fig. 2 we can see that an environmental risk was present from day 1 to 15 for Nord Stream 1 and for the Nord-Stream 2 from Day 1 to 34.

**Figure 3:**
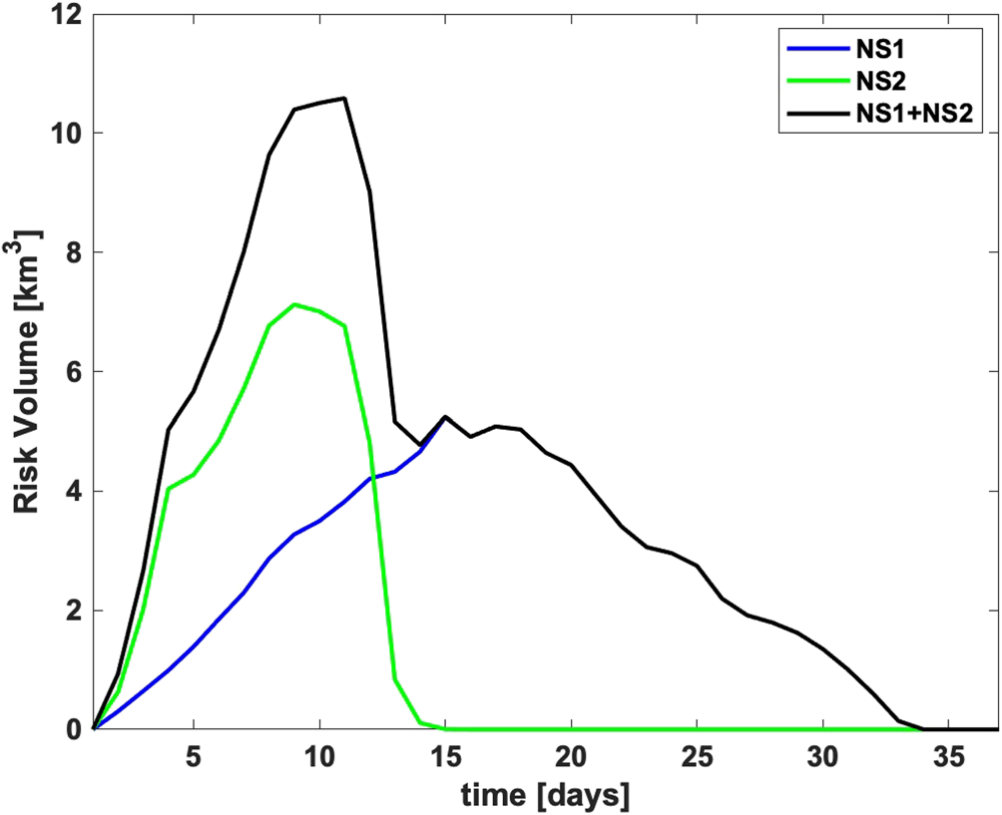
Total volume of water with exceedance of toxicity threshold over time.

The total volume of water with suspended sediment concentrations above the toxicity threshold for each explosion site and aggregate are shown in Fig. 3. The aggregate risk volume reached approximately 11 km^3^ and persisted for 34 days.

**Figure. 4:**
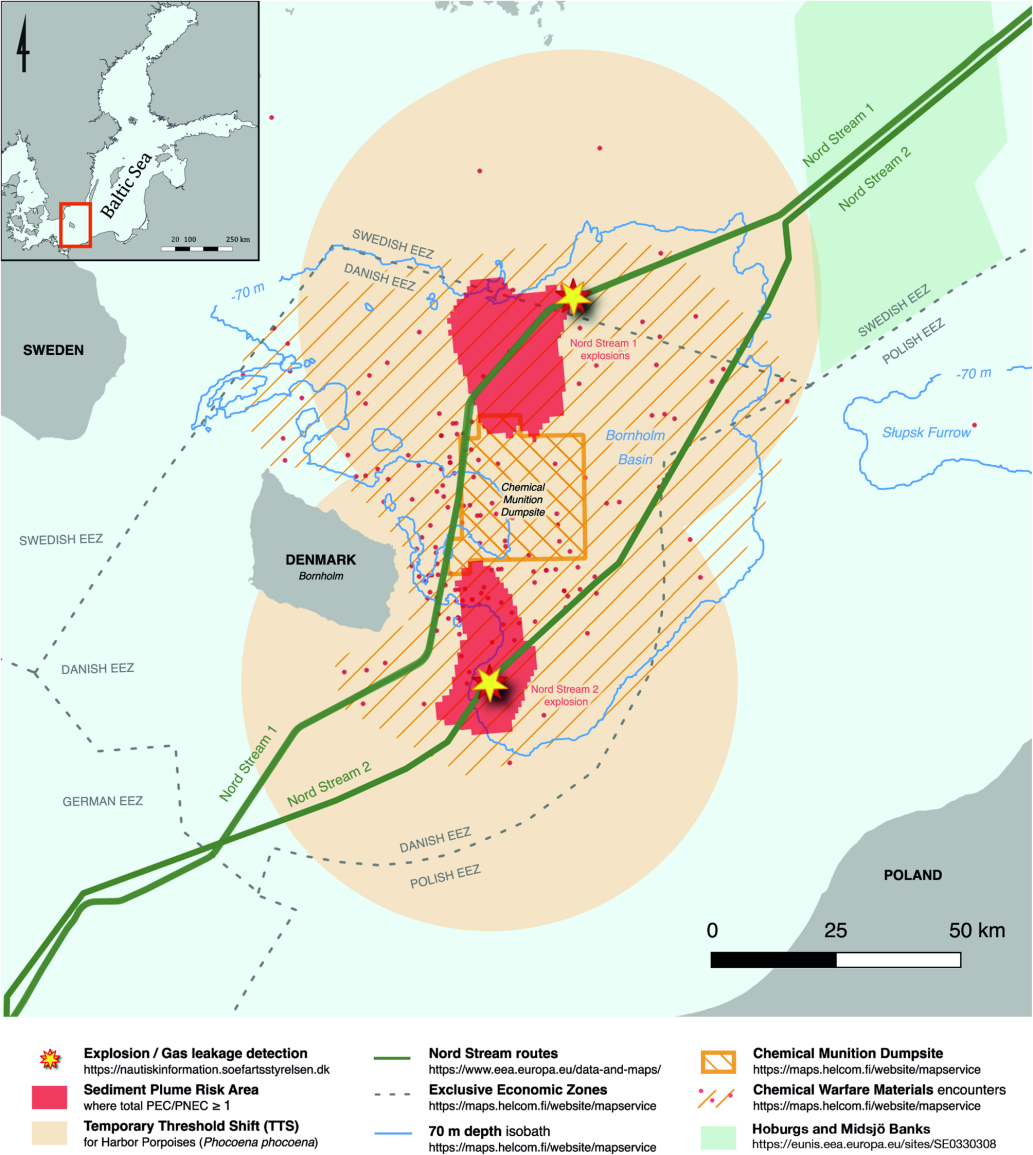
Explosion site map and extent of toxic plume in red (created using QGIS v. 3.28, https://www.qgis.org/it/site/).

Lastly, when we combine the information from the figures in a risk map we can see the total extent of the area at risk in Fig. 4.

The Sediment Plume Risk Areas marked in red is roughly twice size of the area of the island Bornholm (1200 km^2^) where the risk threshold of 5.8 mg sediment/L was exceeded for up to 34 days after the explosions.

The relative toxicity contributions from the different compounds are displayed in Table 2. The relative rank between the three classes of contaminants is metals > organics > CWAs. Metals and organometal compounds (TBT) accounts for > 98% of the total toxicity, with TBT and Pb alone accounts for 75%. For organic pollutants the rank is HBCD > PBDE > Anthracene. For the CWAs rank is As-based > mustard gas related. TBT is a historical antifoulant and the concerning concentrations in the sediment is not surprising in an area with significant shipping activity over decades. TBT is a strong endocrine disrupting compound with a low EQS value that therefore contributes substantially (51.6%) to the total toxicity, despite representing only 0.02% of the total mass of resuspended pollutants. In contrast, Pb was the second most important contributor to the total toxicity due to a relatively high sediment concentration and contributed the most to the total pollutant mass (53%).

## Conclusions

Based on these modelled results we can conclude the following: The Baltic harbour porpoise population is estimated to number about 500 individuals^5^. During breeding season (May–October), this population gathers around the Hoburgs and Midsjö Banks in the Swedish territorial waters^30^, located approximately 40 km east of the northern explosions. It is thus likely that individuals from this population were present in the area in late September and thus could be impacted. Although the low density of porpoises means that the number of individuals impacted was likely low, the population is so small that the loss or serious injury of even a single animal, especially if an adult female, is likely to have an impact on the population^31^. For the Baltic grey seal population and the local Kalmarsund harbour seal population, which are both larger and less vulnerable, the impacts would occur on individual rather than on population level.

The water in Bornholm Deep is characterized by stratification and low vertical mixing. The sites are moreover characterized by low oxygen levels and thus relatively low biological activity. This means that these contaminants have been ‘locked’ away from significant biological exposures while in the unperturbed sediments causing limited environmental risks. The resuspension of CWA contaminated sediments was a major environmental concern during the installation of the Nord Stream 1 and 2 gas pipelines and the reason why they did not take the shortest route through the CWA dump site. The installation was conducted for minimal sediment resuspension, and likely did not cause risks towards the fish community due to release of CWA residues^3^. The rupture of the pipelines and resulting jet of gas did however cause resuspension of 2.5 × 10^5^ metric tons of sediments. The event released historically introduced pollutants to the deepest location of the Bornholm basin and resulted in large volumes of water exceeding the environmental toxic threshold for up to 34 days, which importantly did not reach the surface of the sea nor the surrounding shores. The cause of marine environmental risk was primarily resuspension of TBT and Pb representing ¾ of the total mixture toxicity contributions.

The Bornholm Basin is the traditional spawning and nursery ground for the Eastern Baltic cod (*Gadus morhua*) population. The rupture happened at the end of the normal cod spawning season from March to September. The resuspension of toxic sediments could moreover reach fishes as well as juvenile cods and eggs in the area for more than a month. The most likely long-term impacts on fish would be endocrine disruption due to TBT exposure. Lead (Pb) exposure to fish may induce oxidative stress, affect biochemical and physiological functions among this disrupt neurotransmitters causing neurotoxicity and disruptions to the immune system^32^. The contaminant load resulting from resuspension of sediments by this event likely adds more pressure on already existing ones^33^, putting the e.g. the Baltic cod stock under additional stress. The reproductive success of cod in this area of the Baltic Sea is moreover sensitive to excess turbidity due to resuspended sediments to 1 mg/L causing a risk of the eggs due to sediment adhesion to sink to unfavourable oxygen conditions^34^. The full extent of the impacts caused by the explosions will become more clear in the coming months and years when the stocks of fish and mammals are monitored and assessed from the area.

### Supplementary Information

Supplementary Information.

## Data Availability

The data sets generated regarding the modelling of sediment resuspension in the current study is available from this link: https://oc.iopan.pl/owncloud/index.php/s/6RGnmbLEz2SLwJM. Additional information and data are available in the supporting information section 1–4.
