# Author Correction: Environmental impact of the explosion of the Nord Stream pipelines

**DOI:** 10.1038/s41598-023-48948-y

**Published:** 2023-12-15

**Authors:** Hans Sanderson, Michał Czub, Jaromir Jakacki, Sven Koschinski, Jakob Tougaard, Signe Sveegaard, Torsten Frey, Patrik Fauser, Jacek Bełdowski, Aaron J. Beck, Anna Przyborska, Adam Olejnik, Bogdan Szturomski, Radoslaw Kicinski

**Affiliations:** 1 Department of Environmental Science, Aarhus University, 399 Frederiksborgvej, 4000 Roskilde, Denmark; 2 Department of Hydrobiology, Faculty of Biology, Institute of Functional Biology and Ecology, University of Warsaw, Warsaw, Poland; 3 Institute of Oceanology, Polish Academy of Sciences, Sopot, Poland; 4 Meereszoologie, Kühlandweg 12, 24326 Nehmten, Germany; 5 Department of Ecoscience, Aarhus University, Aarhus, Denmark; 6 GEOMAR Helmholtz Centre for Ocean Research Kiel, Kiel, Germany; 7 Faculty of Mechanical and Electrical Engineering, Polish Naval Academy, Gdynia, Poland

Correction to: *Scientific Reports* https://doi.org/10.1038/s41598-023-47290-7, published online 14 November 2023

The original version of this Article contained an error in the spelling of the author Anna Przyborska which was incorrectly given as Anna Pzyborski.

The original Article has been corrected.

