# Supplementary material for: Environmental impact of the explosion of the Nord Stream pipelines
Source: Sci Rep. 2023 Nov 14;13:19923. doi: 10.1038/s41598-023-47290-7 (PMC10646109; doi:10.1038/s41598-023-47290-7)
Supplement: Supplementary file 1 — Supplementary Information. [file 41598_2023_47290_MOESM1_ESM.docx]

**Supporting Information**

**SI 1: Screening of additives**

Natural gas consists primarily of methane (CH_4_) which is naturally occurring and the exposure to marine organisms is generally low as it rapidly escapes to the atmosphere and has a low inert toxicity towards marine organisms. There are however some impurities in natural gas with methanethiol or mercaptan (CH_3_SH) as the primary one which is added as odorant to the gas in concentrations ranging from 0.016 to 10 parts per million (PPM) in the finished gas (<https://www.gasodorizer.com/mercaptan-inject-natural-gas/>). Mercaptan is very toxic to the aquatic environment with long lasting effects (GHS H400 and H410) (<https://pubchem.ncbi.nlm.nih.gov/compound/methanethiol>), hence the chronic toxicity effect concentration is less than 0.01 mg/L. Hence, we can conservatively assume that 10 PPM of the 115.000 tons natural gas released was mercaptan resulting in approximately 1 ton of mercaptan being released. The mercaptan will generally escape to the atmosphere with the natural gas and not result in noticeable toxicity in the water column with the relatively small amounts.

**SI 2: Release of residues from explosive chemicals (TNT)**:

Two of the most common military explosives are the organic nitroaromatic 2,4,6-trinitrotoluene (TNT) and the cyclic nitramine hexahydro-1,3,5-trinitro-1,3,5-triazine (“Research Department eXplosive”, or RDX) [1]. Of these, TNT is the more toxic compound, classified as GHS hazards H411 (very toxic to aquatic life), H370 (causes damage to organs), and H350 (may cause cancer), among others [2] which is released and mixed in the explosion plume. By definition, high-order detonation of munitions decomposes greater than 99.99% of the organic explosive load to inorganic constituent molecules [3]. In fact, live-fire detonation and blow-in-place detonation (BIP, initiated by an external donor explosive charge) measurements on land show that residual TNT and RDX is closer to 0.0005 – 0.00001% of the original explosive charge ([4, 5, 6, 7]. No similar measurements are available for underwater detonations. Given that the source of oxygen for detonation is the explosive material itself [8], it is likely that underwater detonations have similar efficiency to those on land. Detonation efficiency increases with charge size [3, 7] and for live-fire vs. BIP detonation [9], so under a high-order detonation scenario, the large explosive devices implicated in the pipeline detonations would have produced low levels of residual explosives. In contrast, low-order detonation can leave residue of some 40-50% of the explosive load [6](Taylor et al., 2004). An explosive load of 500 kg undergoing high-order detonation will therefore leave approximately 50 g residual explosive, and a low-order detonation will leave some 200-250 kg of residual explosive. Residual particles from high-order detonations have a very fine diameter (<1 mm) [6]; and are likely to dissolve rapidly [10]. If the residual TNT is rapidly mixed during detonation in the water column within a 50 – 100 m radius, concentrations will be on the order of 20 – 150 ng·L^−1^. These TNT concentrations are approximately 100-fold higher than background concentrations in the adjacent Arkona Basin [11], but are nearly three orders-of-magnitude lower than levels likely to present acute or chronic toxicity risk to aquatic organisms (400 and 30 µg·L^−1^, respectively) [12]. In contrast, TNT release from low-order detonation would result in concentrations between 75 and 600 µg·L^−1^, well above toxicity thresholds. Since, the explosions resulted in the complete breakage of the pipelines we assume that the explosions were of high-order thus leaving no significant toxic residues.

References SI2:

[1] Beck, A. J., Gledhill, M., Schlosser, C., Stamer, B., Böttcher, C., Sternheim, J., ... & Achterberg, E. P. (2018). Spread, behavior, and ecosystem consequences of conventional munitions compounds in coastal marine waters. Frontiers in Marine Science, 5, 141.

[2] ECHA, 2022. European Chemicals Agency, Substance Infocard: 2,4,6-trinitrotoluene. https://echa.europa.eu/substance-information/-/substanceinfo/100.003.900. Accessed 23.11.2022.

[3] Walsh, M. R., Walsh, M. E., Poulin, I., Taylor, S., & Douglas, T. A. (2011). Energetic residues from the detonation of common US ordnance. International Journal of Energetic Materials and Chemical Propulsion, 10(2).

[4] Aurell, J., Gullett, B. K., Tabor, D., Williams, R. K., Mitchell, W., & Kemme, M. R. (2015). Aerostat-based sampling of emissions from open burning and open detonation of military ordnance. Journal of hazardous materials, 284, 108-120.

[5] Pennington, J. C., Hayes, C. A., Yost, S., Crutcher, T. A., Berry, T. E., Clarke, J. U., & Bishop, M. J. (2008). Explosive residues from blow-in-place detonations of artillery munitions. Soil & Sediment Contamination, 17(2), 163-180.

[6] Taylor, S., Hewitt, A., Lever, J., Hayes, C., Perovich, L., Thorne, P., & Daghlian, C. (2004). TNT particle size distributions from detonated 155-mm howitzer rounds. Chemosphere, 55(3), 357-367.

[7] Jenkins, T. F., Walsh, M. E., Miyares, P. H., Hewitt, A. D., Collins, N. H., & Ranney, T. A. (2002). Use of snow-covered ranges to estimate explosives residues from high-order detonations of army munitions. Thermochimica Acta, 384(1-2), 173-185.

[8] US EPA, 1980. 13.3 Explosives Detonation. AP 42, Fifth Edition, Volume I Chapter 13: Miscellaneous Sources. 5 pp.

[9] Hewitt, A. D., Jenkins, T. F., Walsh, M. E., Walsh, M. R., & Taylor, S. (2005). RDX and TNT residues from live-fire and blow-in-place detonations. Chemosphere, 61(6), 888-894.

[10] Lynch, J. C., Brannon, J. M., & Delfino, J. J. (2002). Dissolution rates of three high explosive compounds: TNT, RDX, and HMX. Chemosphere, 47(7), 725-734.

[11] Greinert, J., ed. (2019). Practical Guide for Environmental Monitoring of Conventional Munitions in the Seas-Results from the BMBF funded project UDEMM “Umweltmonitoring für die Delaboration von Munition im Meer” Version 1.1. GEOMAR Report, ISSN Nr.. 2193-8113, DOI 10.3289/GEOMAR_REP_NS_54_2019

[12] Lotufo, G.R., Chappell, M.A., Price, C.L., Ballentine, M.L., Fuentes, A.A., George, R.D., Glisch, E. and Carton, G., 2017. Review and synthesis of evidence regarding environmental risks posed by munitions constituents (MC) in aquatic systems. U.S. Army Engineer Research and Development Center (ERDC) Report: ERDC/EL TR-17-17. 254 pp.

**SI 3: Extrapolation of acoustic impact ranges for the Nord-Stream explosions.**

Fundamental assumption is that the explosions were caused by four charges of 500 kg TNT-equivalent explosives, detonated at the seabed. Ranges at which blast trauma are likely to occur can be estimated based on equations by [1] (SI Figure 1).

**SI Figure 1**: Mammalian damage ranges (Yelverton et al. 1973)

Thresholds applied are from [2] that derive a ‘safe level’ for human divers at 30 kPa·ms. This level is associated with a 10 % risk of injury to lungs or intestines above minor injuries (discomfort/pain, mild internal hemorrhaging). The higher level of 300 kPa·ms is associated with a 50% fatality rate. The distance to the ‘safe level’ was between 4 km and 18 km from the blast site, depending on the depth of the animal. Animals within 1-4 km from the blast site would have been exposed to acoustic impulses above 300 kPa·ms and therefore likely have encountered fatal injuries.

Ranges at which permanent and temporary impact to marine mammal hearing could occur was assessed by estimating the auditory frequency weighted sound exposure levels (SEL) and compare these to thresholds provided [3]. Without actual measurements of the shock wave from the blast and the hydrographical conditions, there is significant uncertainty associated with modelling the propagation of the shock wave from the blasts in a realistic way. Due to this, a similarly uncertain, but much simpler approach can be taken, by extrapolating from actual measurements elsewhere. Most recent and comprehensive measurements are from the North Sea [4]. They provide maximum ranges to permanent hearing loss (PTS) thresholds [3] for harbour porpoises (VHF-cetaceans), dolphins etc. (HF-cetaceans) and baleen whales (LF-cetaceans). These ranges can be extrapolated to a charge weight of 500 kg TNT (SI Figure 2), which results in an impact range for porpoises of 12 km.


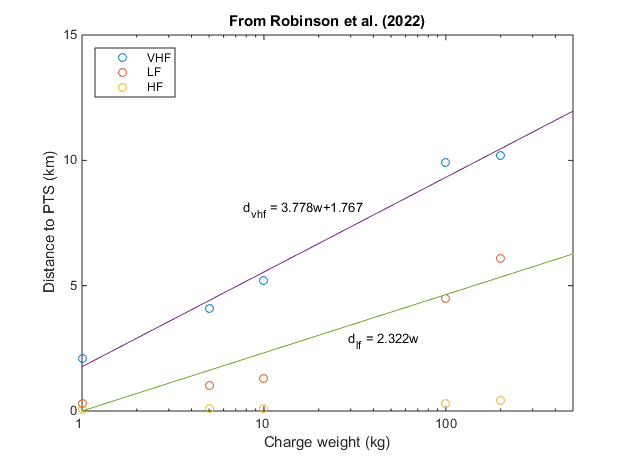


**SI Figure 2**: Relationship between charge weight and distance to effect [4].

As no weighted levels are provided for phocid seals (PCW-weighting), it is difficult to estimate the impact range for harbour seal and grey seal. However, the PCW weighted level at 5.1 km distance [4] is 11 dB below the LF-weighted level (figure 3) and as the PTS-onset threshold [3] for seals is 2 dB higher than for LF-cetaceans, the maximum distance where PTS could be induced must be considerably shorter than the maximum distance of 6 km for baleen whales (LF-cetaceans). The distance can be estimated from Si Figure 4 [4], where the distance where the received level is 13 dB higher than the level at 6 km (PTS distance for LF cetaceans) is estimated to be around 1 km (using the 107 kg charge weight curve).


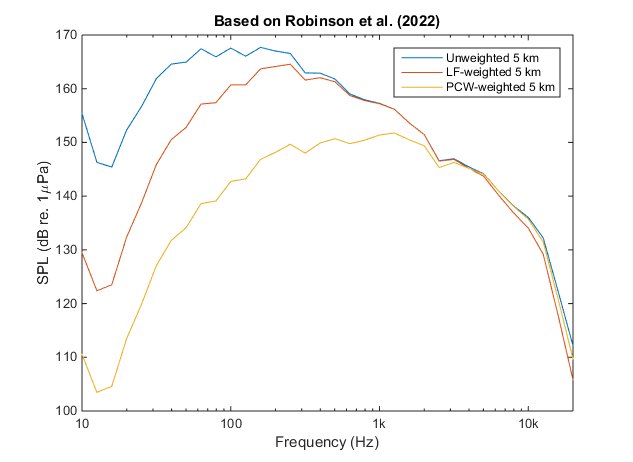


**SI Figure 3a:** PTS distance for LF cetaceans [4]

A precautionary criterion for impact on the hearing is onset of temporary threshold shift (TTS). TTS onset thresholds [3] are 15 dB below the PTS onset thresholds. Maximum ranges for porpoises and seals can therefore be estimated by extrapolating from the distance at minimal PTS-onset distance to the distance where the received level is 15 dB lower (Si Figure 4).


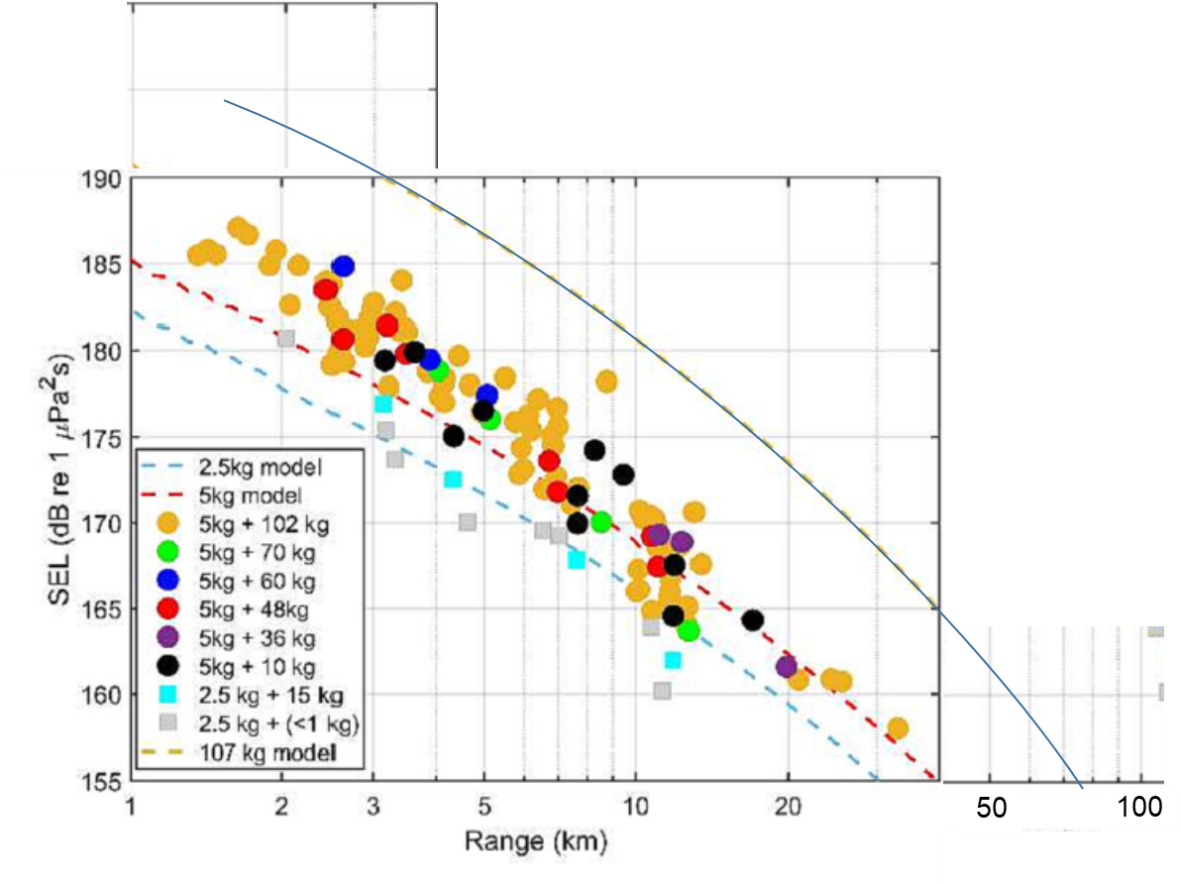


**SI Figure 4:** PTS-onset distance [3]

For porpoises this means extrapolating from 12 km, which is roughly 50 km, whereas for seals the distance extrapolated from 1 km PTS-onset distance is approximately 6 km for maximum distance where minimal TTS could be inflicted. In summary, the following distances of impact were estimated (Table SI Table 1):

**SI Table 1**: Summary of impacts

| Effect | Porpoises | Seals |
| --- | --- | --- |
| Physical trauma | 2-10 km (depth dependent) | 2-10 km (depth dependent) |
| Permanent hearing loss (PTS) | 12 km | ~1 km |
| Temporary hearing loss (TTS) | 50 km | 6 km |

From these figures, it appears that the seals are more vulnerable to trauma in lungs and intestines, whereas porpoises are more prone to permanent and temporary hearing impairment. These distances are associated with considerable uncertainty. Not only is the exact charge size unknown, the location of the explosives unknown (some shielding by the pipeline and/or the seabed could occur), but also the sound propagation conditions not accounted for. The Baltic Sea is very different from the North Sea, because the upper water column is almost fresh water, separated by a strong pycnocline at around 50 m depth. The freshwater means that the absorption of sound is less pronounced than in the North Sea, especially at higher frequencies, which could lead to higher impact ranges and the pycnocline means that sound propagation is complex. However, they correspond well with impact ranges estimated by DW-ShipConsult [5] for detonation of a 263 kg charge in Fehmarn Belt (Western Baltic) and based on thresholds [6]: TTS: 10-50 km, PTS: 3-11 km, trauma: 0.8-4 km.

**References SI3:**

[1] Yelverton, J.T., Richmond, D.R., Fletcher, E.R., Jones, R.K., 1973. Safe distances from underwater explosions for mammals and birds, Albuquerque, New Mexico.

[2] Lance, R.M., Capehart, B., Kadro, O., Bass, C.R., 2015. Human injury criteria for underwater blasts. PLoS One 10, e0143485.

[3] Southall, B.L., Finneran, J.J., Reichmuth, C., Nachtigall, P.E., Ketten, D.R., Bowles, A.E., Ellison, W.T., Nowacek, D.P., Tyack, P.L., 2019. Marine Mammal Noise Exposure Criteria: Updated Scientific Recommendations for Residual Hearing Effects. Aquat. Mamm. 45, 125-232.

[4] Robinson, S.P., Wang, L., Cheong, S.H., Lepper, P.A., Hartley, J.P., Thompson, P.M., Edwards, E., Bellmann, M., 2022. Acoustic characterisation of unexploded ordnance disposal in the North Sea using high order detonations. Mar Pollut Bull 184, 114178.

[5] DW-ShipConsult, 2020. Dokumentation von Minendetonationen Fehmarnbelt - August 2019. Bericht 3/1348-1, Schwentinental, Germany, p. 31.

[6] von Benda-Beckmann, A.M., Aarts, G., Sertlek, H.O., Lucke, K., Verboom, W.C., Kastelein, R.A., Ketten, D.R., van Bemmelen, R., Lam, F.P.A., Kirkwood, R.J., Ainslie, M.A., 2015. Assessing the Impact of Underwater Clearance of Unexploded Ordnance on Harbour Porpoises (*Phocoena phocoena*) in the Southern North Sea. Aquat. Mamm. 41, 503-523.

**SI 4)** **Explosion and sediment resuspension**

An underwater explosion causes an increase in pressure from the point where it occurred within microseconds. In an exothermic self-sustaining chemical reaction high, explosives convert into gaseous reaction at a very high detonation velocity specific to the type of explosive, resulting in a gas bubble. These gases expand much faster than the surrounding matter can respond. The explosion also creates a shock wave in the water and sediment and propagates in all directions. This depends on explosives specific detonation velocity. When a wave front reaches any location, it rapidly raises the pressure from a medium to a maximum value, called a peak positive overpressure, followed by an exponential drop in the surrounding hydrostatic pressure. The period of a continued further pressure drop in the surrounding hydrostatic pressure is called the negative phase period. The mechanism and nature of the explosion are determined by the energetic properties of explosive material as well as the properties of the surrounding area where the detonation takes place such as open or closed area etc. The shock wave generated during the explosion starts to propagate with the detonation velocity of 6,900 m⋅s^-1^, much faster than the sound speed which is approximately 1,450 m⋅s^-1^ in the sea. During its proliferation over many kilometers, it slows down until it reaches the sound speed and then resembles a sound wave.

During an underwater explosion, the sediment containing residues of dumped chemical and conventional munitions is resuspended into the water column. The amount of sediment resuspended depends upon the location of the charge (explosion on the surface, below or above the ground), the density and type of seabed. The volume of ejected sediment from a 500 kg load of TNT placed on the surface of the bottom sediment is approximately 13 m^3^. In addition, the created gas bubbles, moving upwards, introduces a light fraction with a volume equal to about half of its volume into the deep sea (the volume of the bubble in the case of detonation of 500 kg of TNT is in the order of 1000 m^3^). The third factor that influences the resuspension of sediments is the gas leak from the damaged pipelines. Due to its buoyancy, the gas will be transported rapidly to the sea surface. The pressure in the pipeline before explosion was about 105 bar. The rapid pressure drops in the pipeline due to the explosion generated a jet propagating towards the axis of the gas line. Based on [2], the initial medium velocity will be an order of 100 m⋅s^-1^. Assuming, that the resuspension process depends on the speed of the current in the bottom layer of sea, if it exceeds 10 cm⋅s^-1^ (various values can be found in the literature [3] [4]), the area of bottom material that consists of light fraction introduced into the water column under influence of turbulent motion generated by pressure difference between pipe and surrounding medium, has been estimated based on radial profiles of mean axial velocity and velocity along centerline of the jet [5].

The light sediments, because of advection and diffusion processes will be transported as suspended matter (SM). To assess the of development of the SM at the blast sites a 3D hydrodynamic model, coupled to sediment transport model was implemented. The model is based on the MIKE powered by DHI tools which is widely used by the marine research community (for example: [6] [7] [8] [9])The model is based on a flexible, triangular mesh, enabling high spatial resolution in the area of the gas pipeline explosion. The model includes various dynamically coupled modules [10], that includes the mud transport MIKE 3 MT [11] for simulation of the transport, falling and deposition of sediment in the marine. The main core, the hydrodynamic part has been obtained with the MIKE 3 FM HD module [12]. The influence of wind waves is also considered by using the Spectral Wave MIKE 21 FM SW module [13]. The model has been driven based on operational meteorological data that were delivered to the system using an external model covering the entire Baltic Sea area [14], [15]. The initial conditions are based on operational system eBaltic [14] that includes domain created and used for this simulation. The transport and deposition of suspended sediment is related to physical processes such as advection, diffusion and mixing in the water column. The deposition rate depends on size, shape and concentration of the falling particles and the viscosity of the fluid. It increases for the larger and heavier particles as well as and for the less friction.

# References SI4

| [1] | R. Cole, Underwater explosions, Princeton: Princeton University Press, 1948. |
| --- | --- |
| [2] | H. Walden, Mechanika płynów, Warszawa: Wydawnictwa Politechniki Warszawskiej, 1991. |
| [3] | C. Christiansen, K. Edelvang, G. Graf, S. Jahmlich, J. Kozuch, M. Laima, T. Leipe, A. Loffler, L. Lund-Hansen, A. Miltner, K. Pazdro, J. Pempkowiak, G. Shimmield, J. Smith, J. Smith, M. Voss and G. Witt, "Material transport from the nearshore to the basinal environment in the southern Baltic Sea - I. Processes and mass estimates," *Journal of Marine Systems,* vol. 35, pp. 133-150, 2002. |
| [4] | J. Pempkowiak, J. Bełdowski, K. Pazdro, A. Staniszewski, T. Leipe and K. Emeis, "he contribution of the fine sediment fraction to the Fluffy Layer Suspended Matter (FLSM)," *Oceanologia,* vol. 44, pp. 513-527, 2002. |
| [5] | B. CUSHMAN-ROISIN, Environmental Fluid Mechanics, Benoit Cushman-Roisin, 2022. |
| [6] | J. Jakacki, A. Przyborska, S. Kosecki, A. Sundfjord and J. Albretsen, "Modelling of the Svalbard fjord Hornsund," *Oceanologia,* vol. 59, no. 4, p. 473–495, 2017. |
| [7] | A. Przyborska, J. Jakacki and S. Kosecki, "The Impact of the Sopot Pier Marina on the Local Surf Zone," in *In Interdisciplinary Approaches for Sustainable Development Goals*, Berlin/Heidelberg, Springer, 2018, p. 93–109. |
| [8] | Doan Tuan Linh, Trinh Hoang Long, Pham Thanh Van and Vu Van Manh, "Using Mike 21 ST model to assess the sand mining project in Lo river," in *Environmental Informatics and Renewable Energies*, 2013. |
| [9] | E. Zavattero, M. Du, Q. Ma, O. Delestre and P. Gourbesville, "2D Sediment Ttransport Modelling in High Energy River – Application to Var River, France," in *12th International Conference on Hydroinformatics*, 2016. |
| [10] | DHI Software, MIKE 21/3 Coupled Model FM, User Guides, 2008. |
| [11] | DHI Software, "MIKE 21 Flow Model FM, Mud Transport Module," in *MIKE 21 Flow Model FM, Mud Transport Module*, 2013. |
| [12] | DHI Scientific Documentation, ". Hydrodynamic Module," in *MIKE 3 Flow Model FM*, 2011. |
| [13] | DHI Software, "MIKE 21 Wave Modelling," in *MIKE 21 Spectral Waves FM*, 2017. |
| [14] | J. Jakacki, A. Przyborska, A. Nowicki, M. Wichorowski, M. Przyborski, C. Sochacki and R. Tylman, "eBalticGrid - an interactive platform for the visualisation of results from a high-resolution operational Baltic Sea model," *Meteorology Hydrology and Water Management,* vol. 5, no. 2, pp. 13-20, 2017. |
| [15] | J. Jakacki, A. Przyborska and M. Darecki, "Supporting Marine Eenvironment Research by Modelling," *Task Quaterly,* 2018. |
